# Supplementary material for: Molecular Detection of Non-O157 Shiga Toxin-Producing Escherichia coli (STEC) Directly from Stool Using Multiplex qPCR Assays
Source: Microorganisms. 2022 Jan 31;10(2):329. doi: 10.3390/microorganisms10020329 (PMC8878958; doi:10.3390/microorganisms10020329)
Supplement: Supplementary file 1 [file microorganisms-10-00329-s001.zip › microorganisms-1561308-supplementary.pdf]

Table S1. List of all non-top 6 isolates used for inclusivity panel.

Serotype	Number of isolates
O5	4
O8:H14	1
O8:H8	1
O18	1
O22	1
O38	2
O48	1
O55	1
O78	1
O91	2
O113	9
O114	1
O117:H4	5
O117:H7	2
O118: H12	1
O118:H16	1
O118:H30	1
O153	1
O165	2
O174	1
O Undetermined	1
O177	1
OR:H7	2
OR:NM	1
**Grand Total**	44

Table S2. List of all non-top 6 isolates determined by conventional serotyping.

Serotype	Number of isolates
O Rough	3
O Undetermined	15
O5	14
O6	1
O8	1
O11	1
O16	1
O22	1
O27	1
O38	2
O59	1
O69	12
O71	19
O77	1
O80	1
O84	7
O85	2
O86	1

O91	1
O100	2
O108	1
O109	1
O113	1
O118	19
O119	1
O123	2
O128	1
O136	1
O142	1
O146	5
O170	1
O172	1
O175	2
O178	1
O182	2
O183	1
O186	9
O187	1
**Grand Total**	138

Table S3. List of discordant samples.

**Discordant Serotypes Between Stool Molecular Serotype and Conventional Isolate Serotype**	
**Direct Serotype**	**Conventional Serotype**
non-top 6	O103:H2
non-top 6	O103:H2
non-top 6	O103:H2
non-top 6	O103:H2
non-top 6	O103:H25
non-top 6	O103:H25
non-top 6	O121:H19
O103	O71:H8
O121	O26:H non motile
O121	O103:H2
O121	O5:H non motile
O26	O69:H11
O26	O103:H2
O26/O111	O118:H2
**Discordant Serotypes Between Broth Molecular Serotype and Conventional Isolate Serotype**	
non-top 6	O111:H Non-motile
non-top 6	O121:H19
O103	O187:H52
O103	O Undetermined:H2
O103	O Undetermined:H Non-motile
O103	O69:H11
O111	O Undetermined:H Nonmotile
O145	O27:H30
O26	O Undetermined:H Nonmotile
